# Rehabilitation program combining physical exercise and heart rate variability biofeedback in hematologic patients: a feasibility study

**DOI:** 10.1007/s00520-021-06601-2

**Published:** 2021-10-12

**Authors:** Claire Fournié, Chantal Verkindt, Georges Dalleau, Nicolas Bouscaren, Catherine Mohr, Patricia Zunic, Quentin Cabrera

**Affiliations:** 1 Laboratoire IRISSE EA4075, UFR Des Sciences de L’Homme Et de L’Environnement, Université de La Réunion, Le Tampon, La Réunion, France; 2 Centre d’Investigation Clinique, CHU Sud Réunion, Inserm CIC 1410, Saint-Pierre, La Réunion, France; 3 Service d’Hématologie Clinique, CHU Sud Réunion, Saint Pierre, La Réunion, France

**Keywords:** Non-pharmacological intervention, Physical exercise, Heart rate variability biofeedback, Supportive cancer care, Heart rate variability, Hematologic malignancies

## Abstract

**Purpose:**

Hematologic patients have a poorer health-related quality of life due to the disease and its treatments. Non-pharmacological interventions represent an opportunity in tertiary cancer prevention to manage persistent symptoms and support patients in their return to active daily living. This interventional study aimed to evaluate the feasibility of a program combining physical exercise (PE) and heart rate variability biofeedback (HRVB) in hematologic patients.

**Method:**

Hematologic patients in remission within 6 months participated in a 12-week rehabilitation program including 24 supervised sessions of PE associated with 10 supervised sessions of HRVB and daily home-based practice of paced breathing. We assessed patient adherence, fatigue, physical function, and heart rate variability.

**Results:**

Twenty patients were included, 17 completed the protocol and 3 dropped out due to disease progression or time constraints; no adverse events or incidents were reported. Participation rates were 85% for PE and 98% for HRVB-supervised sessions. Significant improvements of physical capacity (6-min walk test, *p* < 0.001; 50-foot walk test, *p* < 0.001), muscle strength (grip force test, *p* < 0.01), and flexibility (toe-touch test, *p* < 0.001; back scratch test, *p* < 0.05) were measured. Coherence ratio (*p* < 0.001) and low-frequency spectral density of HRV signal (*p* < 0.003) increased significantly, suggesting improved autonomic function. Fatigue, static balance, and other time and frequency indicators of HRV were not improved (all *p* > 0.05).

**Conclusion:**

A rehabilitation program combining PE and HRVB is feasible in hematologic patients and effective on physical function. Further research with a larger sample size is needed to investigate effectiveness on patients’ autonomic functions and their impacts on symptomatology.

## Introduction

Hematologic malignancies and their treatment are responsible for serious impairments on overall quality of life [1, 2]. Despite that patients undergoing a hematologic stem cell transplantation usually resume their daily activities within a year of the transplant, most suffer from persistent fatigue, anxiety and depression [2, 3]. Three years after treatment, patients have higher levels of fatigue, dyspnea, and insomnia than the general population [3]. These long-term impairments on quality of life implies that symptom management should continue after treatment cessation to reduce symptom burden and to support patients in their return to active daily living [4].

Physical exercise (PE) is recommended to improve physical function and quality of life during and after treatments in cancer patients [5]. Aerobic reconditioning, resistance training, relaxation, or stretching are well tolerated by hematologic patients [5, 6]. In hematology, PE is effective on cancer-related fatigue, physical function, and quality of life, depending on exercise modalities [7, 8]. Programs that combine moderate-intensity aerobic and resistance trainings (12–14 on the Borg scale corresponding to 70–80% of maximum heart rate), with a duration of over 30 min, 3–5 times per week, for 6–12 weeks, appear to be most effective in patients treated with hematopoietic stem cell transplantation [9].

Heart rate variability biofeedback (HRVB) is a non-pharmacological intervention based on the regular practice of rhythmic breathing at a frequency of approximately 6 cycles/min for which a resonance occurs between cardiac and respiratory rhythms, producing large amplitudes of heart rate variability (HRV) [10]. Although some authors suggested that there is an individual resonance breathing frequency between 4.5 and 7 cycles/min [11], the 6 cycles/min frequency seems adequate to produce a resonance between the cardiac and respiratory systems [12], and the most of the interventional studies in HRVB are based on approximately 6 breaths/min [13]. HRV is used to index autonomic regulation capacity and is generally considered a health indicator [14]. Low variability is a risk factor in all-cause mortality [15], especially for cardiovascular morbidity and mortality [16], and is associated with anxiety [17] and depression [18]. Cancer patients have lower HRV than healthy people, and higher levels of HRV are positively correlated to a better prognosis [19]. According to these authors, the main hypothesis for explaining the lower HRV values in cancer patients than in healthy people is that HRV level is associated with tumor growth through inflammation, oxidative stress, and sympathetic nerve activation, due to disease and aggressive treatments (radiotherapy and chemotherapy). Studies of patients with various chronic illnesses have reported effectiveness of HRVB on HRV increase and autonomic function [20], stress reduction [21], and clinical outcome improvements [22]. Results highlighted the feasibility of HRVB with high rates of participation adherence and satisfaction [21, 22].

The objective of this study was to evaluate the feasibility of a 12-week program combining HRVB and PE in hematologic patients. Feasibility was assessed based on program implementation, adverse events, patient adherence, and level of fatigue. Effectiveness of the two interventions was evaluated based on physical function, measured using a physical test battery, and autonomic function, estimated using HRV parameters. The study was approved by the French ethics committee (NCT03356171).

## Methods

### Participants

Patients diagnosed with various forms of hematological malignancies, in post-treatment remission (full or partial) within the 6 months prior to the study, with stable hemoglobin levels (≥ 9 g/dL), aged 18 to 70 years and French-speaking were included. Patients who at the time of the study were under guardianship, had a medical contraindication to physical exercise, were either anti-arrhythmic or receiving beta-blocker treatment, or experiencing heart failure (left ventricular ejection fraction (LVEF) < 40% measured using electrocardiography pre- or post-therapy) were excluded. All patients provided informed consent.

### Measurements

#### Feasibility, adherence, and fatigue

The feasibility of the program was assessed on the basis of adverse events and potential incidents that occurred during the program; any difficulties encountered in the operational implementation of the program were also identified. Patient adherence was evaluated from participation rates, absences, and their causes.

Fatigue was estimated using a questionnaire (French version) with the Multidimensional Fatigue Inventory (MFI-20) comprising of 20 items divided into 4 dimensions [23]: general/physical fatigue, mental fatigue, reduced activities, and motivation. Each subscale was scored from 0 to 100, with a high score indicating a high degree of fatigue [24].

#### Physical function

Different components of physical function were evaluated using a series of tests that was made from physical tests already performed in cancer patients or the elderly, suggesting that they were feasible and safe for hematologic patients [25–27]. Physical capacity was evaluated using a 6-min walk test (6MWT) in which patients were asked to walk the longest possible distance on a 20-m course [28]. Explosive force was assessed using a 50-foot walk test, which involved timing the distance travelled over a 50-foot round-trip course that participants were asked to complete as fast as possible [25]. Muscle strength was assessed using a grip force test with a JAMAR dynamometer [29]. Stability was tested by a single limb stance test (eyes closed), which is easy to perform and widely used to assess static balance [30]. This test was initially completed with eyes open to prevent injury and anticipate any patient’s inability to perform the test with eyes closed. The flexibility of the posterior muscle chain was assessed by measuring the distance between the middle finger and the ground in forward trunk flexion with straight legs (toe-touch test) [26]. The flexibility of the shoulder girdle was evaluated by measuring the distance between the middle fingers separating the two hands when positioned behind the back, one arm coming from above and the other from below (back scratch test) [26].

#### Heart rate variability

HRV is defined by time fluctuations in the RR interval on an electrocardiogram (ECG) and is measured in both time and frequency domains. The HRV time-domain indices included the standard deviation of normal-to-normal RR intervals (SDNN) and root mean square of successive heartbeat interval differences (RMSSD). The HRV frequency-domain included spectral density components in the very low-frequency band (VLF: 0.003–0.04 Hz), low-frequency band (LF: 0.04–0.15 Hz), high-frequency band (HF: 0.15–0.4 Hz), and their summed total power (TP: 0.003–0.4 Hz) [14]. Absolute values were normalized with a Nepean logarithm (Ln). The coherence ratio was established by identifying the maximum peak in the 0.04–0.26 Hz range, calculating the integral in a window 0.030 Hz wide, centered on the highest peak in that region, which was calculated as follows: (peak power/(TP-peak power)) [31].

HRV was measured using a SymbioLine® non-invasive plethysmograph pulse sensor throughout a 10-min resting period. During HRV recording, patients were seated with knees at 90°, feet flat on the floor, hands on thighs, palms facing upward, and eyes closed, according to standard short-term HRV measurement recommendations [32]. Participants were asked to relax and breathe normally. The last 5 min of the HRV recording were analyzed using HRVanalysis (https://anslabtools.univ-st-etienne.fr) [33].

### Study design and protocol

#### Time assessment

A physical exercise and HRVB instructor monitored patient adherence throughout the program and assessed physical function and HRV at baseline (pre-program) and after the 12-week program (post-program). Hematologists evaluated any adverse events and potential incidents.

#### Physical exercise

The PE program is based on recommendations established in hematological patients [9]. It included 24 supervised sessions of aerobic and resistance training at moderate intensity (12–14 Borg scale corresponding to 70–80% of maximum heart rate [34]) twice a week. Each session included a 5-min warm-up of soft joint movements, 30-min of aerobic exercises (walking or step aerobics), 20-min of resistance circuit-training, and a 5-min cool-down. Inspired by a treadmill walking protocol already tested in hematologic patients [35], an outdoor walking protocol was used, consisting of alternating periods of walking at a comfortable or accelerated speed with periods of rest. Gradually, rest periods were reduced and walking periods were increased to up to 30-min of continuous walking, alternating between normal (7–9 Borg scale) and accelerated speeds (10–13 Borg scale) without rest. The step aerobics was based on movements choreographed to music, stimulating aerobic capacity, physical coordination, and balance [36]. Resistance training consisted of circuit-training workouts involving main muscle groups (arm curl, squats, plank, modified curl-up) with body weight, dumbbells (1–2 kg), or medicine balls (3–5 kg). Particular attention was paid to posture, compliance to the moderate intensity threshold, and progressivity according to the individual’s abilities throughout the program. This program followed the recommendations of the American College of Sports Medicine [5] and specific recommendations for hematologic patients [9].

#### Heart rate variability biofeedback

HRVB consisted of 10 supervised 1-h sessions over 12 weeks (approximately weekly) and 20-min of daily home practice as recommended by Lehrer’s protocol [37]. Biofeedback was carried out with Symbiofi® cardiac coherence software (SymbioCenter® technology, SymbioLine® Professional, SYMBIOFI, Loos, France) which provides numerous interactive exercises to learn how to control breathing at a specific rate of 6 breaths/min. For example, coherence state was illustrated with a stormy weather (low coherence) or a sunny weather (high coherence) displayed on the screen. The patients were asked to try to control their respiration so as to reach a coherence state, thereby keeping the images of sunny weather on their screen for as long as possible. A respiratory guide, coherence score, and real-time HRV recordings using a plethysmograph pulse sensor (tachogram) were displayed on the screen to help patients achieve a cardiac coherence state. Coherence scores indicate coherence in the HRV signal; a high score is reached when heart rhythm pattern becomes sine wave-like at a frequency of approximately 0.1 Hz. Concerning daily practice, patients used a light metronome (DODOW®, LIVLAB® technology, Paris, France) to help them control their breathing at 6 breaths/min without biofeedback. Patients were required to record their daily practices in a logbook monitored by the instructor.

### Statistical analysis

Data were collected on a confidential and anonymous database. Qualitative variables were expressed as numbers and percentages, and quantitative variables were expressed as mean ± SD. The comparison between pre- to post-program was evaluated with a paired Student’s *t*-test on R software version 1.2.5033 [38].

## Results

### Study sample, adhesion, and adverse events

This study included 20 patients, 17 completed the protocol and 3 dropped out due to relapse (2) or time constraints (1). Baseline characteristics of the patients are presented in Table 1. The PE participation rate was 85% (20.5 ± 3.6 of the 24 scheduled sessions); and the HRVB participation rate was 98% (9.8 ± 0.5 of the 10 scheduled supervised sessions). For daily home practice, patients completed 71.67 ± 21.2 sessions over the 12-week rehabilitation program. Patients reported no adverse events or incidents along the protocol.

**Table 1:** Baseline characteristics of the study sample

Parameters	All (*n* = 20)	Withdrawn (*n* = 3)	Full protocol (*n* = 17)
Gender (M/F)	11/9	2/1	9/8
Age (years)	54.1 ± 11.0	51.7 ± 6.0	54,5 ± 11,7
Leukemia ***n*** (%)	5 (25%)	1 (33%)	4 (24%)
Lymphoma ***n*** (%)	5 (25%)	1 (33%	4 (24%)
Myeloma ***n*** (%)	10 (50%)	1 (33%)	9 (52%)
No transplant ***n*** (%)	6 (30%)	1 (33%)	5 (29%)
Autologous transplant ***n*** (%)	11 (55%)	1 (33%)	10 (59%)
Allogenic transplant ***n*** (%)	3 (15%)	1 (33%)	2 (12%)
Complete remission ***n*** (%)	13 (65%)	1 (33%)	12 (71%)
Partial remission ***n*** (%)	7 (35%)	2 (67%)	5 (29%)
BMI (kg/m^**2**^)	25.3 ± 4.5	24.6 ± 2.5	25.4 ± 4.8
Systolic BP (mmhg)	140.1 ± 25.3	139.0 ± 37.3	140.2 ± 24.2
Diastolic BP (mmhg)	83.1 ± 15.3	79.3 ± 8.1	83.8 ± 16.4

*Mean* ± *SD unless stated otherwise*

### Effects on fatigue

None of the subscales of the MFI-20 changed during the intervention (Table 2; all *p* > 0.05) despite a trend towards decreasing for general/physical fatigue (− 10.78 ± 22.2, *p* = 0.062) and mental fatigue (− 11.52 ± 24.0, *p* = 0.065).

**Table 2:** Pre- and post-program fatigue questionnaire results (MFI-20)

Fatigue subscales (MFI-20)	Pre-program	Post-program	Change (Δ)	*p*
General/physical fatigue	32.68 ± 23.1	21.90 ± 25.2	− 10.78 ± 22.2	0.062
Mental fatigue	31.62 ± 19.3	20.10 ± 22.2	− 11.52 ± 24.0	0.065
Reduced activities	31.86 ± 25.6	25.98 ± 31.3	− 5.88 ± 27.1	0.385
Reduced motivation	12.50 ± 14.0	17.65 ± 19.8	5.15 ± 18.8	0.275

*Mean* ± *SD. Pre****-****/post-program comparison with paired Student’s t-test*

### Effects on physical function

A significant improvement in physical function was measured between the beginning and end of the program (Table 3) with an increase in distance travelled during 6-min (TM6) of + 100.41 ± 45.6 m (*p* < 0.001**), and a reduction in time for the 50-foot walk test of − 1.51 ± 1.2 s (< 0.001**), indicating an augmented walking speed. Muscle strength (grip force test) significantly increased on the right hand (+ 1.41 ± 2.0 kg, *p* = 0.010*) and on the left hand (+ 2.65 ± 2.6 kg, *p* < 0.001**). Flexibility was also significantly improved; we recorded decreases in distance to the ground (toe-touch test) of − 8.06 ± 8.1 cm (*p* < 0.001**) and distance between the two hands (back scratch test) with the right hand overhead of − 2.65 ± 4.2 cm (*p* = 0.018*). Nonetheless, static balance and back scratch test with the left hand overhead did not improve over time (*p* > 0.05).

**Table 3:** Pre- and post-program physical tests results

Tests	Pre-program	Post-program	Change (Δ)	*p*
6-min walk test *m*	495.88 ± 88.6	596.29 ± 83.6	+ 100.41 ± 45.6	< 0.001**
50-ft walk test *s*	9.19 ± 2.6	7.68 ± 1.7	− 1.51 ± 1.2	< 0.001**
Grip force test (R) *kg*	27.18 ± 6.6	28.59 ± 6.6	+ 1.41 ± 2.0	0.010*
Grip force test (L) *kg*	25.00 ± 6.1	27.65 ± 6.8	+ 2.65 ± 2.6	< 0.001**
Single limb stance test (R) *s*	7.56 ± 4.6	10.14 ± 10.7	+ 2.58 ± 8.5	0.230
Single limb stance test (L) *s*	13.70 ± 13.3	15.47 ± 14.6	+ 1.78 ± 10.4	0.492
Toe-touch test *cm*	8.47 ± 20.4	0.41 ± 15.7	− 8.06 ± 8.1	< 0.001**
Back scratch test (R) *cm*	3.53 ± 13.8	0.88 ± 14.1	− 2.65 ± 4.2	0.018*
Back scratch test (L) *cm*	11.06 ± 12.1	8.35 ± 11.8	− 2.71 ± 8.3	0.196

*Mean* ± *SD. Pre-/post-program comparison with paired Student’s t-test*

### Effects on heart rate variability

Results (Table 4) show that SDNN and RMSSD did not change over time (*p* > 0.05) despite upward trends of SDNN (+ 4.70 ± 18.3). Similarly, spectral density in TP (Ln(TP)) did not change despite an upward trend of + 0.40 ± 1.2 (*p* > 0.05). Both spectral density in VLF (Ln(VLF)) and HF (Ln(HF)) did not change (*p* > 0.05) over time. In contrast, spectral density in LF (Ln(LF)) increased significantly at post-program (*p* = 0.003*) with an improvement of + 1.16 ± 1.3. Coherence ratio also increased significantly with an improvement of + 17.88 ± 13.8 (< 0.001*).

**Table 4:** Pre- and post-program HRV parameters results

HRV parameters	Pre-program	Post-program	Change (Δ)	*p*
SDNN	35.84 ± 22.8	40.54 ± 20.9	+ 4.70 ± 18.3	0.305
RMSSD	25.04 ± 15.9	25.24 ± 12.4	+ 0.20 ± 10.9	0.941
Ln (TP)	6.70 ± 1.3	7.07 ± 1.0	+ 0.40 ± 1.2	0.223
Ln (VLF)	5.61 ± 1.8	5.60 ± 1.1	− 0.02 ± 1.3	0.962
Ln (LF)	4.99 ± 1.6	6.15 ± 1.6	+ 1.16 ± 1.3	0.003*
Ln (HF)	4.94 ± 1.5	4.79 ± 1.1	− 0.15 ± 1.0	0.541
Coherence ratio	55.00 ± 12.8	72.88 ± 16.0	+ 17.88 ± 13.8	< 0.001**

*Mean* ± *SD. Pre-/post-program comparison with paired Student’s t-test*

## Discussion

Both PE and HRVB were feasible and appreciated by patients. Results show a high participation rate for both PE (85%) and HRVB (98%) intervention, and an average practice of 6 HRV sessions per week at home during the intervention. This rehabilitation program combining PE and HRVB was able to proceed according to the terms of the protocol without any difficulties related to its implementation. Although some barriers to patient adherence were intentionally removed, particularly those related to physical limitations, there were still individual constraints related to disease progression and time/availability. In future post-treatment interventions, supervised programs via video conferencing could be offered to make it more convenient for patients and their individual schedules.

Fatigue levels did not deteriorate during the intervention, highlighting good patient tolerance to the program (Table 2), despite large inter-individual variability. PE is known to improve fatigue status in cancer patients [7, 39], and we could have expected a significant reduction in fatigue subscales at post-program. Although our results showed a non-significant decreasing trend for mean scores of general/physical fatigue, mental fatigue, and reduced activities, these results remain encouraging because of a mean change higher than the estimated minimal clinically important difference (MCID) of 3 points (Table 2), and our small sample size. In order to more accurately assess the effects on fatigue status, we could precisely measure hemoglobin levels at pre- and post-program to take into account its possible confounding effect. Indeed, it has already been shown that a low hemoglobin level was associated with high level of fatigue and reciprocally a high hemoglobin level was associated with low level of fatigue in hematological cancer patients [40].

Results highlight the program’s effectiveness on physical function with a significant improvement in physical capacity, muscle strength, and flexibility (Table 3). The physical capacity results evaluated with the 6MWT scores show a pre-post-program increase greater than the minimal clinically important difference (MCID) which was estimated from 25 m in cardiac patients [41]. Another study in cardiac patients showed that performance during the 6MWT correlated with maximum aerobic capacity (VO2 peak), all the more so when VO2 peak is low [42], suggesting the importance of improving 6MWT scores in these patients. Given the physical deconditioning of hematologic patients within 6-month post-treatment, we assume that physical deconditioning of cardiac and hematologic patients is comparable. Although the increases in grip force test results reflected an increase in overall muscle strength [29], scores were inferior than the estimated MCID between 5.0 and 6.5 kg [43]. Studies have shown that muscle strength can be considered a marker of survival in elderly cancer patients [44], highlighting the importance of rehabilitation programs for strength recovery in hematologic patients. Improved flexibility shows a gain in patient mobility, which should promote a more active lifestyle. Although our results should be qualified by the absence of a control group, they are consistent with other studies evaluating the effects of PE in hematologic patients [8, 45]. Improvement of physical function, although it could be partly attributed to spontaneous recovery, proves that the PE program was well-adapted to the patients’ functional capabilities and did not generate excessive fatigue.

Regarding the program’s effectiveness on HRV, we observed no significant improvements in either global variability characterized by SDNN and TP, or in vagal tone represented by RMSSD and HF spectral power [14]. However, HRVB produced a peak power in LF band corresponding to high synchronization between cardiac and respiratory systems when respiration rates were approximately 6 breath/min, which corresponds to vagal tone modulations on the sino-atrial node [10, 31, 32]. Therefore, the increase in both LF band and coherence score (Table 4) suggests that patients were able to achieve cardiac coherence state and could reflect a possible mobilization of vagal regulation at rest. Other studies using HRVB in patients with various chronic illnesses provide similar results with respect to an increase in the LF band of the HRV signal, which the authors interpreted as an increase in vagal tone [46–50]. Although results regarding SDNN and TP are inconclusive, the feasibility of HRVB in hematologic patients encourages the testing of a larger sample to determine the effectiveness of the rehabilitation program on HRV. The high inter-individual variability in cancer patients [51] could explain the absence of significant increases of SDNN and TP in our study, especially since other results showed an increase in HRV after programs of both physical exercise [52] and HRVB [53].

In conclusion, a rehabilitation program combining PE and HRVB is feasible in hematologic patients and effective on physical function. However, further research with larger sample sizes and randomized controlled study designs is needed to investigate effectiveness on patients’ autonomic function. In addition, impacts on patient symptomatology, post-treatment recovery, and quality of life should be studied to better understand the challenges of rehabilitation programs based on non-pharmacological interventions such as PE and HRVB.

## Data Availability

The datasets generated during and/or analyzed during the current study are available from the corresponding author on reasonable request.
